# Acute Optic Neuritis: An Update on Approach and Management

**DOI:** 10.18502/jovr.v18i4.14556

**Published:** 2023-11-30

**Authors:** Sepideh Jamali Dogahe, Parastou Pakravan, Mohammad Pakravan

**Affiliations:** ^1^Department of Ophthalmology, Mayo Clinic, Rochester, MN, USA; ^2^Bascom Palmer Eye Institute, University of Miami, Miami, FL, USA; ^3^University of Texas Medical Branches, Galveston, TX, USA; ^6^Mohammad Pakravan: https://orcid.org/0000-0002-9708-2916

**Keywords:** MOG, Neuromyelitis optica, Optic neuritis

## Abstract

This review discusses the physical examination and diagnostic tests necessary to diagnose optic neuritis (ON) and provides an update on the approach and management of acute ON. A comprehensive search of the PubMed database was conducted, limited to English-language journals and recent publications. A total of 160 articles were initially screened by title, of which 73 articles were included in the narrative synthesis.

ON is an inflammation of the optic nerve that can be caused by different systemic and neurological disorders. It is commonly presented as a subacute unilateral painful vision loss, and based on its clinical manifestation, it can be classified as typical or atypical. Atypical ON is bilateral with visual acuity of worse than 20/200 or has an atypical demographic presentation for demyelination, such as a non-Caucasian male with optic disc swelling, for which neuromyelitis optica spectrum disorder (NMOSD), myelin-oligodendrocyte glycoprotein antibody-associated disease (MOGAD), or other etiologies should be considered. Steroids and immunosuppressants are the main treatment options for ON, and timely treatment initiation is critical to preventing irreversible vision loss, especially in atypical cases.

##  INTRODUCTION

Optic neuritis (ON) is an inflammation of the optic nerve and is the most common optic neuropathy affecting young adults. ON may present in the setting of different systemic and neurological disorders such as multiple sclerosis (MS), neuromyelitis optica spectrum disorder (NMOSD), and myelin-oligodendrocyte glycoprotein antibody-associated disease (MOGAD).^[1]^ Early differentiation of MS, NMOSD, and MOGAD is critical, as each has a different pathophysiology that affects treatment options, clinical outcomes, and morbidities.

Typically, ON is presented as a subacute unilateral painful vision loss Based on its clinical presentation, ON can be classified as typical or atypical, that is, MS-associated or non-MS-associated, respectively.^[2]^ Children under the age of 10 are more likely to present with bilateral ON. In older children, however, ON appears similar to that in adults and is mostly unilateral.^[3]^

The etiology of ON is unclear, but multiple mechanisms for inflammation of the optic nerve have been proposed;^[4,5]^ of which autoimmunity is the most suggestive pathophysiology. Other causes include infections,^[6,7,8]^ postvaccinations,^[9,10]^ autoimmune diseases such as sarcoidosis, Bechet's disease, systemic lupus erythematosus, granulomatous diseases, paraneoplastic disorders, toxins, or drug toxicities.

Steroids and immunosuppressants are the main treatment options for ON, and especially in atypical cases, the timely onset of therapy is critical to prevent irreversible vision loss.^[11]^ This review aims to describe the recent findings on the etiology, classification, diagnosis, and management of ON.

### Data Collection

This article provides an update on the approach and management of acute ON. We limited the PubMed literature search to English-language journals and included keywords such as “optic neuritis", “Neuromyelitis Optica", “Neuromyelitis Optica (NMO) Spectrum Disorder", “NMO Spectrum Disorder", “Optic Nerve Disease", “Optic Neuropathy", “NMOSD", “MOGAD", “ON". A total of 160 articles were initially screened by title, and 73 articles were ultimately included in the narrative synthesis.

### Clinical Features and Presentation

Typical ON mostly affects Caucasians and women between the ages of 18 and 45 and presents as a monocular, subacute loss of vision associated with or without orbital pain, worsening of eye movements.^[12,13]^ Patients may experience varying severity of vision loss, ranging from mild fogginess to no light perception (NLP). Vision usually diminishes within the first two weeks and may improve even without any treatments. Phosphene-like flashes of light may sometimes be experienced.^[14]^

The Caucasians seem to be at a higher risk for ON due to the higher incidence of MS in this population. African or Caribbean patients, on the other hand, are more likely to have an atypical pattern of ON that necessitates corticosteroid therapy more often compared to Caucasians.^[15]^ Additionally, Moss et al suggested that race/ethnicity may have a significant impact on contrast sensitivity and visual acuity in affected eyes in their study with African Americans having worse outcomes.^[16]^ Furthermore, the etiology and management of ON may differ based on the patient's ethnicity.^[17]^ Women are more likely to suffer from ON than men, with a female-to-male ratio of approximately 3:1 in MS and 7:1 in neuromyelitis optica (NMO).^[18]^

In children, demyelinating ON can occur alone or in conjunction with MS, acute disseminated encephalomyelitis (ADEM), or as part of an autoantibody response to either AQP4 (NMOSD) or MOG (MOGAD).^[19]^ Also, children younger than 10 years of age are more likely to present with bilateral ON and more likely to have a prodromal illness. While older children are more likely to present with unilateral ON,^[20]^ which is associated with a greater likelihood of MS later in their lives.^[21]^ Seizures, motor symptoms, irritability, or systemic findings may accompany ON, which necessitates ruling out the ADEM.

ON is considered atypical when it presents bilaterally, with visual acuity of worse than 20/200, or with atypical demography for demyelination such as in a non-Caucasian male with optic disc swelling, for which NMOSD, MOGAD, or other etiologies should be considered. In NMOSD, myelitis can develop concurrently, later, or rarely before ON. If the site of involvement is more posterior, vision loss may be painless. On the other hand, MOGAD may present with severe pain because it mainly involves the sheath.^[22,23]^ In case of a recurrent ON, one should think of NMOSD, MOGAD, and CRION. Whether there is pain or not, recurrent ON in a MOG-IgG antibody-positive patient is characterized as chronic relapsing inflammatory optic neuritis (CRION), which is another subtype of ON.^[24]^ Sarcoidosis^[25,26,27,28]^ with or without uveitis and lung involvement, tuberculosis^[23][24][25,26,27,28][29][30][31]^, and syphilis^[32,33,34]^ are less frequent etiologies.

Color vision and contrast sensitivity deficits are commonly seen in ON; visual field (VF) defects are usually central. Examination of color vision and VF is crucial for both the diagnosis and the prognosis.^[35,36]^

The Uhthoff phenomenon^[37]^ which is a transient worsening of neurological symptoms, usually a consequence of body temperature elevation, can be seen with ON. Drinking cold water can alleviate the severity of this phenomenon. Flu-like symptoms may also accompany visual symptoms in ON.^[38]^

### Physical Exam Findings

Since visual acuity can range from normal to NLP, contrast sensitivity testing may be a better indicator of visual function loss. However, it is not always readily available, and color vision tests with Ishihara or Hardy-Rand-Rittler can be a better option as contrast and color function diminish in parallel. The red and green desaturation tests are prone to error due to their subjective nature. Brightness comparison is another alternative and supportive test. Relative afferent pupillary defect (RAPD) should be checked like in any other unilateral or asymmetric neuropathy. Utilizing a 0.3 log unit neutral density may be helpful in detecting a subtle RAPD.

A dilated fundus exam is necessary, which can be normal in two-thirds of cases or may reveal disc swelling.^[39]^ Disc swelling is another feature of atypical ON and mandates a more extensive workup. Extensive hemorrhage around the disc is more likely to be seen in MOGAD.^[40,41,42]^ Several weeks after the attack, optic atrophy may be detected. When optic atrophy is present at the time of acute event, compressive optic neuropathy should be ruled out.

Atypical clinical findings include age > 50 years old, bilateral presentation, lack of pain or progressive vision loss and pain over weeks, vitreous cells, optic disc pallor at presentation, optic disc swelling, retinal hemorrhage and exudate, lack of vision recovery, and the presence of other systemic symptoms such as hiccups and vomiting. Saccadic dysmetria, 6 ${}^{th}$ nerve palsy, and internuclear ophthalmoplegia (INO) can also be seen with ON. In the event of these findings, an extensive workup is warranted.

### Diagnostic Procedures:

Ancillary testing

### • Visual field (VF) test

VF analysis is helpful to locate and quantify visual impairment. VF may demonstrate different patterns and severity based on the etiology of ON. A higher rate of non-central scotoma may be observed in NMOSD-ON compared to demyelinating ON; altitudinal and hemianopia field defects may also be characteristic features of NMOSD patients.^[43]^ Foveal threshold testing can assess the amount of central vision loss and a large target size can be requested if visual acuity is poor.

### • Magnetic resonance imaging (MRI)

MRI of the orbits and brain with and without gadolinium and fat suppression is routinely used to evaluate patients with ON who are experiencing their first episode of demyelinating disease. After intravenous gadolinium, fat-suppressed T1 weighted MRI can detect ON by enhancing the optic nerve; however, a normal MRI necessarily does not rule out the demyelinating disease. In the absence of disease, the optic nerve sheath could still be mildly enhanced.^[44]^

In a typical ON, a small segment of the intra-orbital optic nerve is usually involved, whereas NMOSD and MOGAD involve more extensive and posterior segments of the optic nerve or even a chiasma. Additionally, optic nerve sheath edema and inflammatory swelling of the surrounding tissue can exist, which indicates extensive optic nerve damage in MOGAD patients.^[45]^ Atypical ON usually does not demonstrate periventricular white matter lesions as MS does, but brainstem abnormalities in the area postrema may be detected. Diagnosis of ON in adults and children with brain MRI abnormalities can increase the risk of MS in the future.^[46,47,48]^

### • Optical coherence tomography (OCT)

In the acute phase of retrobulbar ON, the retinal nerve fiber layer (RNFL) is normal or slightly thickened. RNFL is markedly thickened when optic nerve swelling presents. After the acute phase is resolved, and in about three months, RNFL becomes thin, especially in NMOSD. RNFL of the contralateral uninvolved eye of MS-related ON may reveal thinning compared to controls; however, it is usually normal in NMOSD. Prior ON can accurately be detected using OCT, especially by measuring the ganglion cell inner plexiform layer (GCIPL) thickness.^[49,50,51]^ Thinning of GCIPL may reveal earlier loss compared to RNFL.

### • Visual evoked potential 

The visual evoked potential can be affected even when the clinical involvement of ON is subtle and a decrease in amplitudes and a delay in latencies may be detected in patients with ON.^[52]^

### Other Tests:

AQP4-IgG or NMO antibodies and antibodies directed against myelin oligodendrocyte glycoprotein (MOG) are two of the tests that have recently gained more attention. It is important to consider testing AQP4-IgG- and MOG-IgG-associated disease in all adults with isolated ON, especially in Asian populations as well as in pediatric isolated ON, to differentiate MS from NMOSD or MOGAD. MOG-ON and AQP4-ON exhibit similar levels of GCIPL and RNFL thinning, but AQP4-ON has significantly worse visual outcomes.^[53]^ MOG-IgG-positive patients may be more likely to relapse than AQP4-IgG-positive patients, causing damage accumulation whereas patients with AQP-4-IG have more severe ON episodes.^[54]^ A study showed RNFL is better preserved in the eyes of patients with MOG-IgG antibodies compared to those with AQP4-IgG antibodies following ON as well as better visual outcomes. In patients with AQP4-IgG positive ON, RNFL thickness was significantly lower (*P* = 0.023). Another finding of this study was a significantly lower final mean VF defect in AQP4-IgG-positive ON patients (*P* = 0.046).^[55]^

### Differential Diagnoses

Differential diagnoses of ON include ischemic optic neuropathy, compressive optic neuropathy, NMOSD, Leber's hereditary optic neuropathy, MOGAD, inflammatory optic neuropathy (sarcoidosis, granulomatosis with polyangiitis), toxic/metabolic, infectious (neuroretinitis, Lyme disease, syphilis, tuberculosis), medication-induced optic neuropathy, infiltrative, idiopathic, and big blind spot syndromes. There is a possibility of post-COVID ON as a neurological complication. A number of case reports in the literature discuss optic neuropathy as a manifestation of COVID infection or following COVID vaccination. In these cases, patients were reported to develop a gradual loss of vision one to two weeks after severe COVID infection unilaterally or bilaterally; females tended to suffer from ON and retinal complications.^[56,57,63]^

### Treatment and Prognosis 

Optic neuritis treatment trial (ONTT) was a multicenter, randomized, prospective clinical trial that was designed to evaluate the efficacy and safety of intravenous methylprednisolone (IVMP) (I gr/day for three days) followed by 11 days of oral prednisolone (1 mg/kg/day), which was compared to oral prednisolone alone and placebo. IVMP led to a more rapid recovery of vision but did not improve the final visual acuity.^[64]^ Based on ONTT, intravenous or oral corticosteroids did not have any beneficial effects on visual acuity and VF after six months. However, patients who received low-dose oral prednisolone experienced twice the number of early recurrences within six months when compared to the controls.^[65]^ Patients who received high-dose IVMP had fewer MS relapses within the first two years but not afterward.^[66]^ ONTT did not evaluate the efficacy of high-dose oral prednisolone; however, later studies with smaller sample sizes suggested it as an alternative. In NMO patients, a significant decrease in Expanded Disability Status Scale (EDSS) scores were observed exclusively in individuals with a history of prior oral corticosteroid use (*P* = 0.026) before IVMP administration. Moreover, reduced EDSS scores showed significance in patients without a history of previous immunosuppressant use, while this significance was not apparent among those who had utilized immunosuppressants in the past (*P* = 0.023).^[67]^ However, it should be emphasized that 1 mg/kg of oral prednisolone can increase the likelihood of recurrence and should be avoided.

ON can occur in MS, NMOSD, and MOGAS. Distinguishing between these three conditions is crucial because it can significantly impact the choice of treatment and overall clinical outcomes.^[68]^ Prompt initiation of IVMP in atypical ON is more beneficial in terms of visual outcomes and should be started, especially in NMOSD, MOGAD, and sarcoidosis.^[69,70]^ More aggressive treatments such as plasmapheresis or IVIG could be considered if there was no vision improvement after steroid therapy. In such cases, comanagement with the neurology and internal medicine teams is highly recommended. Although ONTT suggested steroid therapy as an optional measure, it should be considered in any case of atypical ON when the underlying cause is yet to be determined. This scenario is often the case for a patient with his/her first episode of ON. In an inpatient setting, it is better to administer IVMP 500–1000 mg/day for 3–5 days so that its adverse effects can be detected more easily and ancillary tests can be done more quickly.^[64]^

In case of a typical ON and a negative systemic workup, the patient should be referred to a neurologist to consider disease-modifying therapies after steroid treatment. IVIG, plasmapheresis, and administration of recently FDA-approved monoclonal antibodies including eculizumab, inebilizumab, and satralizumab are different measures that are suggested for the treatment of NMOSD.^[68]^

According to ONTT, more than 88% of cases in the three groups ended up with visual acuity of equal or more than 20/40 in 6 months. However, contrast sensitivity and color perception may remain poor despite visual acuity recovery. The IV steroid group showed faster vision recovery but a similar outcome.^[64]^ In atypical ON including NMOSD and MOGAD, visual outcome is different and quick initiation of treatment is crucial for the improvement of outcome which is generally guarded. Since NMOSD has a poor clinical prognosis, early treatment with high-dose corticosteroids and plasma exchange can help in acute attack.^[68]^ NMOSD treatment later than five days can significantly worsen the prognosis. Additionally, plasmapheresis can be effective for acute treatment which is more beneficial if received within 7 days of onset of NMOSD-associated ON and may lead to better outcomes compared to delayed treatment.^[71,72]^ Patients should continue long-term immunotherapeutic agents, such as rituximab or other FDA-approved monoclonal antibody treatments. Additionally, in the case of NMOSD, it is crucial that it is differentiated from MS because the treatment options differ and some MS therapies such as IFN-β, natalizumab, and oral fingolimod, can even worsen NMO.^[73]^ ON in MOGAD, however, generally had better visual outcomes and responds better to corticosteroids. Sometimes, these patients are corticosteroids-dependent or have recurrent relapses that would require them to be on long-term IVIG or tocilizumab.

There have been multiple clinical trials investigating newer drugs to treat ON in NMOSD and MOGAD. Three new FDA approved drugs are introduced for the treatment of ON in NMOSD, including eculizumab, inebilizumab, and satralizumab. Rozanolixizumab and satralizumab are currently being investigated as treatment options for ON in MOGAD.^[68]^ In case of CRION, the patient needs to be on long-term low-dose corticosteroids. Immunosuppressants like azathioprine, methotrexate, cyclophosphamide, or mycophenolate have been beneficial for inducing long-term remissions.^[74]^ Thus, it should be emphasized that atypical ON prognosis is quite different and usually worse than typical demyelinating ON. Prompt initiation of treatment with a multidisciplinary strategy is required. Assistance of neurology and sometimes infectious disease/internal medicine teams are warranted for improvement of visual outcome and prognosis.

In conclusion, this study sheds light on the complexity of ON and emphasizes the importance of early diagnosis and tailored management. The diverse etiologies, clinical presentations, and diagnostic approaches highlighted in this research underline the need for a multidisciplinary approach involving ophthalmologists, neurologists, and other specialists. By staying updated on the latest advancements in diagnostic tools and treatment options, healthcare professionals can strive for better outcomes and preservation of visual function in patients with ON.

##  Financial Support and Sponsorship

None.

##  Conflict of Interest

None.
